# Assessment of patients with acute respiratory symptoms during the COVID-19 pandemic by Telemedicine: clinical features and impact on referral

**DOI:** 10.31744/einstein_journal/2020AO6106

**Published:** 2020-11-25

**Authors:** Tarso Augusto Duenhas Accorsi, Karine De Amicis, Alexandra Régia Dantas Brígido, Deborah de Sá Pereira Belfort, Fábio Cetinic Habrum, Fernando Garcia Scarpanti, Iuri Resedá Magalhães, José Roberto de Oliveira Silva, Leon Pablo Cartaxo Sampaio, Maria Tereza Sampaio de Sousa Lira, Renata Albaladejo Morbeck, Carlos Henrique Sartorato Pedrotti, Eduardo Cordioli

**Affiliations:** 1 Hospital Israelita Albert Einstein São PauloSP Brazil Hospital Israelita Albert Einstein, São Paulo, SP, Brazil.

**Keywords:** Telemedicine, Coronavirus infections, COVID-19, Respiratory tract infections, Pandemics, Referral and consultation

## Abstract

**Objective:**

To characterize variables associated with referral to the emergency department following Telemedicine consultation during the COVID-19 pandemic.

**Methods:**

Cross-sectional retrospective study conducted between March and May 2020, with a sample of 500 adult patients. The inclusion criterion was the manifestation of respiratory symptoms, regardless of type.

**Results:**

The mean age of patients was 34.7±10.5 years, and 59% were women. Most patients (62.6%) perceived their own health status as malaise and some (41.4%) self-diagnosed COVID-19. Cough (74.4%), rhinorrhea (65.6%), sore throat (38.6%) and sneezing (20.6%) were the most common infection-related symptoms. Overall, 29.4% and 16% of patients reported dyspnea and chest pain, respectively. The Roth score was calculated for a sizeable number of patients (67.6%) and was normal, moderately altered or severely altered in 83.5%, 10.7% and 5.6% of patients, respectively. The percentage of suspected COVID-19 cases was 67.6%. Of these, 75% were managed remotely and only one quarter referred for emergency assessment.

**Conclusion:**

Telemedicine assessment is associated with reclassification of patient's subjective impression, better inspection of coronavirus disease 2019 and identification of risk patients. Referral is therefore optimized to avoid inappropriate in-person assessment, and low-risk patients can be properly guided. Telemedicine should be implemented in the health care system as a cost-effective strategy for initial assessment of acute patients.

## INTRODUCTION

The coronavirus disease 2019 (COVID-19) pandemic has turned Telemedicine into a key resource for the health care system, due to its ability to safely serve large populations via prompt actions in a cost-effective manner.^(^^1^^)^ In Telemedicine, anamnesis and discreet physical examination (observation through a camera, physician-guided self-examination and assessment of vital signs using home medical devices) play a vital role in the identification of red flags in patients with acute respiratory symptoms.^(^^2^^)^ Telemedicine may also help oligosymptomatic patients to follow appropriate guidelines at home.^(^^3^^)^ It should be noted that many cases in China were due to in-hospital transmission.^(^^4^^)^ Appropriate measures must be undertaken to ensure that the individuals with low-risk diseases (especially those who are not suspected of COVID-19) are isolated from high-risk individuals, and to limit their consumption of health resources. It is equally important to minimize the spread of infection among health care professionals and control community transmission. Telemedicine has an amazing potential in both cases.^(^^1^^)^

Given the rapid global spread of COVID-19, several publications describing the clinical characteristics of the disease and discussing different treatment strategies have came out. However, supportive treatment is the only available alternative to date, regardless of disease stage.^(^^5^^)^ In developing countries, most suspected cases are not tested for diagnostic confirmation, except inpatients. Therefore, underreporting is common.^(^^6^^)^ However, regardless of testing, clinical identification of suspected COVID-19 cases is possible and may be used to provide appropriate guidance to low-risk patients in order to avoid in-person emergency department visits.^(^^7^^)^

In spite of theoretical benefits, few studies have investigated the role of Telemedicine in patient referral due to acute respiratory symptoms during the pandemic.^(^^2^^)^ To fill in this gap in the medical literature, this study set out to characterize the demographic and clinical profiles of patients seeking care by Telemedicine, due to acute respiratory symptoms during the pandemic. Emergency department referrals were quantified and associated variables analyzed.

## OBJECTIVE

To characterize variables associated with referral to the emergency department following Telemedicine consultation during the coronavirus disease 2019 pandemic.

## METHODS

### Study design and participants

This study was approved by the local institutional review board (CAAE: 31106820.5.0000.0071, ≠ 4,001,939). All patients accessed Telemedicine services provided by *Hospital Israelita Albert Einstein* (HIAE), in São Paulo (SP, Brazil) via mobile application and spontaneously requested medical assessment. Between March and May 2020, 500 adult patients (≥18 years of age) were retrospectively and randomly enrolled. Patients seeking care due to respiratory tract symptoms of any kind (acute or progressive cough, expectoration, dyspnea, chest pain, rhinitis or sore throat), or suggestive of infection (fever, chills, sweating, myalgia, fatigue, malaise, anorexia, headache, and others) were included. Suspicion of COVID-19 was based on presentation of at least one symptom of each kind. The primary endpoint was emergency department referral. Eligible patients were defined as those with complete clinical data (subjective impressions, complaints and respective duration, history of current illness and past medical/surgical history), as well as description of physical examination findings. Patients with COVID-19-related infection or connection problems were excluded.

### Roth score

Eligible patients were instructed to perform self-examination and the expiratory maneuver in order to obtain the Roth score. The calculation of Roth score involves instructing patients to take a deep breath and try to count out loud from 1 to 30 before exhaling. After this procedure, patients are asked to take three calm deep breaths and the test is repeated. Time is counted by the examiner. The second test is the valid one. In this study, three scoring levels were considered based on the highest number reached and the time elapsed, as follows: normal (>10 and >7 seconds); moderately altered (7 to 10 and/or 5 to 7 seconds), and severely altered (<7 and/or <5 seconds). Correlation of Roth scores and oximetry corresponds to normal (>95%), moderately altered (90% to 94%) and severely altered (<90%), respectively.^(^^8^^)^

### Statistical analysis

This was an observational study based on a cohort of randomly selected patients. Descriptive statistics was used to analyze patient clinical profile. Continuous variables were expressed as medians and standard deviation. Categorical variables were expressed as counts and percentages. There were no missing data. Multiple and bivariate logistic regression was performed in order to investigate correlations between reported and collected variables and the primary endpoint. P values <0.05 were indicative of significant differences. The confidence interval (CI) adopted was 95%.

## RESULTS

All patients enrolled in the study (500) were analyzed. The female sex prevailed in the study population (59%) and the mean age was low (34.7±10.5 years). Patients sought Telemedicine services within a mean of 4.67±4.82 days after onset of symptoms and, in most cases, this was their first medical appointment, with no prior virtual or in-person emergency department visit. Regarding pre-existing comorbidities potentially associated with poor prognosis for COVID-19 disease, chronic lung diseases were the most prevalent (8.2%), followed by hypertension (6.2%), obesity (5.0%), diabetes (2.4%, with 0.6% of patients reporting insulin use) and heart disease (2.2%). Few patients reported smoking (2.2%), immunosuppression (0.6%) or chronic kidney disease (0.2%). Cerebrovascular disease was not reported, and the percentage of pregnant women in the sample was small.

A high percentage of patients (62.6%) reported subjective negative perception of their own clinical status, and some (41.4%) reported self-diagnosed COVID-19 disease. Despite the pandemic, almost one-fifth of patients reported having cold (14.8%), influenza (7.6%) or pneumonia (2.6%). Some patients reported suspicion of mild upper airway infections, such as sinusitis (7.8%) or pharyngitis (4.4%), and 21.4% did not mention any specific diagnosis. The major concern that triggered the selection of Telemedicine services was the possibility of contracting COVID-19 infection (43%), followed by fear of referral to the emergency department (7.6%). Concerns related to hospital admission, community transmission and death were reported by 6.4%, 6.2% and 0.6% of patients, respectively ( Table 1 ).

**Table 1:** Subjective patient self-analysis

Variable		n (%)
Question: how do you feel?		
	Well	187 (37.4)
	Bad	313 (62.6)
Question: what disease do you think you have?		
	I do not know	107 (21.4)
	COVID-19	207 (41.4)
	Flu	38 (7.6)
	Cold	74 (14.8)
	Pneumonia	13 (2.6)
	Sinusitis	39 (7.8)
	Pharyngitis	22 (4.4)
Question: what do you fear most regarding your current condition?		
	None	171 (34.2)
	COVID-19	215 (43)
	Admission	32 (6.4)
	Death	3 (0.6)
	In-person assessment	38 (7.6)
	Transmission to others	31 (6.2)
	Other	10 (2)

Cough was the most commonly reported respiratory symptom (74.4%). It was described as dry or productive cough with clear discharge in 87.4% and 11.2% of cases, respectively. Only 1.4% of patients reported purulent sputum. Rhinorrhea was another common symptom (65.6%), while sore throat (38.6%) and sneezing (20.6%) were the less prevalent. Two manifestations recently associated with COVID-19 infection were described: anosmia (24.8%) and dysgeusia (24.6%). Of patients with infectious symptoms, 45% had fever, 44.8% myalgia or arthralgia, 41.4% fatigue, 40.6% headache and 24% digestive tract symptoms, such as nausea, vomiting and diarrhea. Dyspnea and chest pain, which are commonly associated with disease severity, were reported by 29.4% and 16% of patients, respectively. Half of these patients perceived dyspnea as mild symptom, and only a small number of patients reported severe dyspnea ( Table 2 ).

**Table 2:** Clinical manifestations

Variable		n (%)
Cough		372 (74.4)
Mucus/discharge		
	No	437 (87.4)
	Watery	56 (11.2)
	Yellow, green, brown	7 (1.4)
	Nasal congestion	217 (43.4)
Rhinorrhea		
	No	9 (1.8)
	Yes	328 (65.6)
	Watery	157 (31.4)
	Yellow, green, brown	6 (1.2)
Sneezing		103 (20.6)
Sore throat		193 (38.6)
Adenomegaly		14 (2.8)
Hoarseness		45 (9)
Dyspnea (subjective)		147 (29.4)
Anosmia		124 (24.8)
Dysgeusia		123 (24.6)
Intensity of subjective dyspnea		
	No	351 (70.2)
	Mild	73 (14.6)
	Moderate	63 (12.6)
	Severe	13 (2.6)
Chest pain		80 (16)
Nausea/vomiting		45 (9)
Diarrhea		75 (15)
Fatigue		207 (41.4)
Myalgia or arthralgia		224 (44.8)
Headache		203 (40.6)

As for the possibility of conducting physical examination via Telemedicine, apart from use of a camera, some patients used home medical devices to check vital signs. Some were instructed to perform self-examination and the expiratory maneuver required to obtain the Roth score. Approximately 9.4% of patients accessing Telemedicine services reported fever, whereas sweating, conjunctival inflammation and adenomegaly were uncommon. Heart rate was measured in 214 patients, with a mean of 82.5±14.2. In contrast, blood pressure and oximetry were measured in only 31 and 16 patients, respectively, and not thought to very be useful. Overall, Roth score was calculated for an expressive number of patients (67.6%) and was normal, moderately altered and severely altered in 83.5%, 10.7%, and 5.6% of patients, respectively ( Table 3 ). The Roth score of all 147 patients self-reporting dyspnea was calculated. However, changes were limited to 48 (32.6%) cases.

**Table 3:** Physical examination

Variable		
Fever		47 (9.4)
Sweating		4 (0.8)
Adenomegaly on self-examination		12 (2.4)
Conjunctival inflammation		7 (1.4)
Roth score		
	Not determined	183 (36.6)
	NormaI (nc >10 and time >7 seconds)	265/317 (83.5)
	Moderate altered (nc=7-10 and/or time=5-7 seconds)	34/317 (10.7)
	Severe altered (nc <7 and/or time <5 seconds)	18/317 (5.8)
Heart rate, n=214		82.5±14.2
Systolic blood pressure, n=31		134.2±22.4
Diastolic blood pressure, n=31		86.8±12.6
Oximetry, n=16		96.5±2

Results expressed as n (%) or mean±standard deviation.

nc: number of counting in a single expiration.

The percentage of suspected COVID-19 cases was 67.6%. Of these, 75% were managed remotely, whereas precisely one quarter was referred for immediate emergency assessment. The number of suspected COVID-19 cases increased by approximately 50% after Telemedicine assessment relative to patient self-diagnosis. The following variables were independently correlated with referral to emergency department: female sex (odds ratio *–* OR: 1.91; 95%CI: 1.01-3.63; p=0.047), heart disease (OR: 14.28; 95%CI: 2.45-83.07; p=0.003), lung disease (OR: 2.49; 95%CI: 1.02-6.07; p=0.044), subjective dyspnea (OR: 3.48; 95%CI: 1.76-6.88; p<0.001), chest pain (OR: 11.34; 95%CI: 5.43-23.71; p<0.001), suspected COVID-19 cases (OR: 2.84; 95%CI: 1.32-6.09; p=0.007), and moderately or severely altered Roth score (OR: 33.49; 95%CI: 8.43-133.15; p<0.001) ( Table 4 ).

**Table 4:** Suspected COVID-19 cases and correlation with referral

Suspected COVID-19 cases, n (%)	338 (67.6)			
Referral to emergency department, n (%)	125 (25)			
Correlation of variable with referral to emergency department	OR	95%CI		p value
		Lower	Upper	
Sex female	1.91	1.01	3.63	0.047
Heart disease	14.28	2.45	83.07	0.003
Lung disease	2.49	1.02	6.07	0.044
Dyspnea (subjective)	3.48	1.76	6.88	<0.001
Chest pain	11.34	5.43	23.71	<0.001
Diagnosis J11 (ICD-10)	2.84	1.32	6.09	0.007
Dyspnea (Roth score)				
Normal	1.86	0.83	4.19	0.132
Moderate or severe	33.49	8.43	133.15	<0.001

Bivariate logistic regression.

OR: odds ratio; 95%CI: 95% of confidence interval; ICD-10: International Classification of Diseases.

## DISCUSSION

Virtual communication is a multipurpose tool to deal with the burden imposed by COVID-19 on the health care system. Technological advancements are providing increasing access to healthcare in cases involving geographical separation of patients and providers. Effective assessment via smartphone audio and video has become increasingly feasible, making Telemedicine a potentially universal resource.^(^^9^^)^ Aside from safety, comfort and ease of access, this simple tool is able to offer optimal high-value care. Especially in cases of acute viral infections with no specific ongoing treatment, patients can be effectively examined via remote assessment.^(^^1^^,^^2^^)^ Yet, in spite of several theoretical advantages, the implementation, regulation and physician acceptance of Telemedicine has been slow, as has the accumulation of evidence of effectiveness.^(^^10^^)^ However, the COVID-19 pandemic led to an increase in Telemedicine availability, and quickly demystified its limitations, allowing enhanced collection of scientific data to support its implementation.^(^^11^^)^ During pandemics, referral of symptomatic high-risk patients for emergency care is key. Preventing patients who are not suspected of COVID-19 or suffer from low-risk diseases from inappropriately seeking in-person care is also important to prevent patient infection and community transmission, spare healthcare professionals and avoid inappropriate use of resources. Finally, correct therapeutic guidance and management of the huge burden of instant daily news is vital. These measures are even more crucial in developing countries.^(^^9^^)^

The number of daily visits to the Telemedicine Department of HIAE increased 16-fold and the geographic coverage was also expanded. This service is already being regulated and ensures safety, comfort and well-being of both, medical staff and patients. Easy access via mobile app allows prompt inexpensive medical consultation around the clock. By the end of the data collection period in this study, São Paulo had 44,411 confirmed cases of COVID-19 infection, 3,600 deaths, 10,000 hospital admissions and 85% intensive care unit bed occupancy rate.^(^^12^^)^ The intrinsic limitations of Telemedicine often overestimate referral. Any red flag in history taking or physical examination is worthy of in-person investigation. The major question raised by this study is whether this type of medical evaluation could actually be effective in reducing emergency department visits.

This study was based on a limited sample of 500 patients with acute respiratory symptoms, who did not have a confirmed diagnosis of COVID-19 disease. Tested patients with confirmed COVID-19 disease diagnosis were excluded, since they had already sought medical care and received appropriate guidance and follow-up. Also, in a study involving 92 patients with confirmed COVID-19 diagnosis evaluated by Telemedicine, Khairat et al., reported a reduction in the number of emergency department visits.^(^^13^^)^ Of the total number of patients, a sizeable proportion of those seen in outpatient visits (mainly low-risk patients) can be effectively managed remotely.^(^^14^^)^ In theory, Telemedicine allows providers to screen patients to avoid visits to the emergency department. Patients can be directed to an urgent care clinic, outpatient follow-up, or local COVID-19 testing site instead.^(^^7^^)^ However, there are no published data regarding Telemedicine use in this population in particular.^(^^3^^,^^10^^,^^11^^)^

Mann et al., reported a large expansion of Telemedicine care after the beginning of the pandemic, and 56% urgent assessments of confirmed or suspected COVID-19 cases, but referral data were not provided.^(^^10^^)^ As in the study by Khairat et al.,^(^^13^^)^ the population in this investigation comprised primarily young adult women. This may have reflected the fact that younger individuals are more familiar with technological devices, whereas older patients undergoing regular medical follow-up tend to communicate directly with healthcare staff, instead of using Telemedicine services, or tend to seek in-person assistance in more severe cases. This sample comprised primarily low-risk patients reporting respiratory symptoms during the pandemic.

Almost all clinical features of COVID-19 disease described to date refer to hospitalized patients, presumably the more severe cases, with no large outpatient case series and no case descriptions involving Telemedicine.^(^^4^^,^^15^^)^ Studies reporting suspected COVID-19 cases among patients presenting respiratory symptoms during the pandemic are also lacking. In this study, almost one-third of the patients had a poor prognosis regarding COVID-19 infection-related comorbidities. According to health guidelines, these patients should seek medical care. Additionally, the 62.6% of patients who subjectively classified their own general health status as poor presumably would seek health care services. This finding alone supports the benefit of the initial medical evaluation via Telemedicine. History revealed a wide range of respiratory symptoms, and COVID-19 infection was suspected by the medical team in 67.6% of cases, increasing the rate of self-diagnosis. Regardless of emergency department referral, most patients had already benefited from Telemedicine guidance regarding behavior in cases suspected of COVID-19. Chest pain and dyspnea were reported by 16% and 29.4% of patients, respectively. The fact that such symptoms are much more difficult to assess using Telemedicine makes chest pain of any kind a major trigger of emergency department referrals.

Increasing efforts are being made to expand the use of remote physical examination. Dermatology, ophthalmology and neurology examination can be easily performed.^(^^16^^)^ Telemedicine allows the observation of runny nose and cough (dry or productive), as well as the assessment of mental status, facial expression of pain, prostration, sweating, hyperemia, cyanosis or pallor. Wheezing may be detected sometimes. Respiratory distress and rate, interrupted speech, use of accessory muscles, nasal flaring and see-saw breathing are relatively easy to identify. Vital signals may also be checked using self-examination (guided or not) and home medical devices (thermometer, sphygmomanometer, watch, frequency meters, oximeter, mobile phone apps etc.). The oropharynx can be examined by correctly positioning the camera, and erythema, exudate and tonsil changes may be detected. Enlarged lymph nodes may be palpated via physician-guided self-examination.^(^^2^^,^^17^^)^ However, the most important evaluation is the determination of pulmonary involvement. Dyspnea is associated with potentially life-threatening disease.^(^^18^^)^ In this study, self-reported dyspnea was significantly associated with referral to ED. However, the definition of dyspnea is subjective and multifactorial pathophysiology makes determination of etiological diagnosis and symptom severity challenging. A systematic review concluded that there is no gold standard method for clinical assessment of dyspnea. Therefore, ancillary tests are mandatory.^(^^19^^)^ Telemedicine assessment of dyspnea is even more complex. Most patients in this sample were not diagnosed as dyspnea based on clinical observation. However, this does not rule out oxygenation or ventilation disorders. Few patients provided oximetry data. Overall, 317 patients were evaluated based on the Roth score (number counted in and/or duration of a single breath), which can be associated with different pulse oximetry cutoffs. This score can be used to determine the occurrence and severity of dyspnea in Telemedicine. The Roth score is often employed in cases of dyspnea or red flags detected on clinical evaluation. Of 317 patients, 83.6% had normal expiration values and were managed conservatively. In contrast, moderately or severely altered Roth scores were significantly associated with referral to emergency department. Application of the Roth score led to reclassification of almost two-thirds of self-reported dyspneic patients. Therefore, Roth scores were associated with changes in medical approach in most cases requiring the use of this tool.

In this study, exactly one quarter of patients were referred for the emergency department and 75% were managed remotely. Cases suspected of COVID-19 based on medical team observations were associated with emergency department referral to ensure access to treatment for potentially more serious conditions. Severe comorbidities (chronic lung and heart conditions) and self-reported chest pain were also associated with referral, since comorbidities may predispose to severe infection and chest pain may be a manifestation of thrombotic events or pulmonary complications.^(^^4^^,^^14^^)^ These findings suggest high-risk patients may indeed be easily identified by remote assessment. Remaining patients represented the biggest challenge to be overcome by Telemedicine. In spite of the high number of patients with self-perceived serious conditions, history and physical examination (including Roth scores) allowed the reclassification of most patients as low-risk. Self-reported dyspnea was also associated with patient referral and represents a limitation of remote physical assessment. However, some dyspneic patients were not referred based on clinical judgment and the Roth score. Normal scores were negatively associated with emergency department referral, and only a few patients with moderate to severe changes were referred.

The solutions to some unique challenges posed by an outbreak of infectious disease must be incorporated into Telemedicine.^(^^1^^)^ The impact of the COVID-19 outbreak on the Italian health care system could have been less significant had Telemedicine been widely used in that country. A subjective perception of lack resistance on the part of the patients has been reported.^(^^10^^,^^14^^)^ Also, the overwhelming burden imposed by the COVID-19 pandemic may lead to health care provider burnout. The implementation of Telemedicine may therefore ensure the efficiency of the health care system.^(^^20^^)^

This is a significant moment in virtual health care and probably the biggest ever of global health care transformation expected to occur in the near future. Apart from the comfort and safety provided by this type of service, there is growing scientific evidence to support the effectiveness of Telemedicine for referral of high-risk patients and guidance of low-risk patients. With the exception of physical examination, Telemedicine should offer the same quality of care as in-person visits.^(^^17^^)^ Several remote physical examination strategies can be used to add value to history and home medical devices capable of transmitting relevant information will eventually be incorporated. This study revealed that Telemedicine was able to properly refer high-risk patients and reclassify and redirect low-risk patients during the pandemic, thereby reducing volunteer seeking of emergency services.

However, this study has some limitations. First, it was an observational retrospective study. Second, the diagnosis of COVID-19 was not confirmed. Thirdly, although the Roth score was often used, the correlation between Roth score and desaturation has not been externally validated. Finally, referred patients were not followed. However, the fact that this study reflects a real-life experience, in which 500 patients sought virtual medical services due to a very common condition was thought to be a strong point.

## CONCLUSION

With regard to patients with acute respiratory symptoms during the pandemic, Telemedicine assessment was associated with reclassification of patients’ subjective impressions, better inspection of COVID-19 and identification of risk patients. Virtual medical evaluation optimized patient referral, avoiding inappropriate in-person assessment and allowing appropriate guidance of low-risk patients. Remote Roth score application should be encouraged. Telemedicine may be incorporated to the health care system as a cost-effective strategy tool for initial assessment of acute patients.
