# Supplementary material for: Assessment of patients with acute respiratory symptoms during the COVID-19 pandemic by Telemedicine: clinical features and impact on referral
Source: Einstein (Sao Paulo). 2020 Nov 25;18:eAO6106. doi: 10.31744/einstein_journal/2020AO6106 (PMC7690926; doi:10.31744/einstein_journal/2020AO6106)
Supplement: Translation

# Translation: Avaliação de pacientes com sintomas respiratórios agudos durante a pandemia de COVID-19 via Telemedicina: características clínicas e impacto no encaminhamento

**Authors:** Tarso Augusto Duenhas Accorsi, Karine De Amicis, Alexandra Régia Dantas Brígido, Deborah de Sá Pereira Belfort, Fábio Cetinic Habrum, Fernando Garcia Scarpanti, Iuri Resedá Magalhães, José Roberto de Oliveira Silva, Leon Pablo Cartaxo Sampaio, Maria Tereza Sampaio de Sousa Lira, Renata Albaladejo Morbeck, Carlos Henrique Sartorato Pedrotti, Eduardo Cordioli

**Affiliations:** 1 São PauloSP Brasil Hospital Israelita Albert Einstein, São Paulo, SP, Brasil.

**Keywords:** Telemedicina, Infecções por coronavírus, COVID-19, Infecções respiratórias, Pandemias, Encaminhamento e consulta

## Abstract

**Objetivo:**

Caracterizar as variáveis associadas ao encaminhamento à emergência após consulta de Telemedicina durante a pandemia de COVID-19.

**Métodos:**

Estudo transversal e retrospectivo, realizado entre março e maio de 2020, com amostra de 500 pacientes adultos. O critério de inclusão foi apresentação de sintomas respiratórios, independente do tipo.

**Resultados:**

A média de idade dos pacientes foi de 34,7±10,5 anos, e 59% eram do sexo feminino. A maioria dos pacientes (62,6%) se classificou subjetivamente como tendo um mal-estar, e alguns (41,4%) autodiagnosticaram COVID-19. Tosse (74,4%), rinorreia (65,6%), dor de garganta (38,6%) e espirros (20,6%) foram os sintomas mais comuns relacionados à infecção. Dispneia e dor torácica foram relatados por 29,4% e 16% dos pacientes, respectivamente. Foi calculado o escore de Roth de um número considerável de pacientes (67,6%), obtendo resultado normal em 83,5%, moderadamente alterado em 10,7% e grave em 5,6%. A percentagem de casos suspeitos de COVID-19 foi de 67,6%, e 75% desses foram gerenciados remotamente, com apenas um quarto sendo encaminhado para avaliação imediata na emergência.

**Conclusão:**

A avaliação da Telemedicina está associada à reclassificação da impressão subjetiva do paciente, melhor inspeção da COVI-19 e identificação de pacientes de risco. O encaminhamento é otimizado, para evitar avaliação presencial inadequada, e permite que os pacientes de baixo risco sejam orientados de forma apropriada. A Telemedicina deve ser implementada no sistema de saúde como estratégia com boa relação custo-efetividade para a avaliação inicial de pacientes agudos.

## INTRODUÇÃO

A pandemia causada pelo novo coronavírus (COVID-19) elevou a Telemedicina ao patamar de recurso essencial para o sistema de saúde, devido a sua capacidade de prestar atendimento a populações numerosas, com segurança, por meio de ações imediatas e com boa relação custo-efetividade.^(^^1^^)^Na Telemedicina, a anamnese e o exame físico discreto (observação por uma câmera, autoexame guiado pelo médico e aferição de sinais vitais com dispositivos médicos de uso doméstico) têm papel fundamental na identificação de sinais de alarme em pacientes com sintomas respiratórios agudos.^(^^2^^)^Além disso, a Telemedicina pode ajudar pacientes oligossintomáticos a seguir diretrizes adequadas em casa.^(^^3^^)^Cabe notar que muitos casos na China estavam relacionados à transmissão intra-hospitalar.^(^^4^^)^Medidas apropriadas devem ser adotadas para garantir que indivíduos com doenças de baixo risco – sobretudo aqueles sem suspeita de COVID-19 – sejam isolados de indivíduos de alto risco e consumam poucos recursos de saúde. A minimização da disseminação da infecção entre os profissionais da saúde e o controle da transmissão comunitária são igualmente importantes. A Telemedicina tem um potencial impressionante em ambos os casos.^(^^1^^)^

Dada a rápida disseminação global da COVID-19, surgiram diversas publicações descrevendo as características clínicas da doença e discutindo diferentes estratégias de tratamento. Entretanto, o tratamento de suporte é a única alternativa disponível até o momento, independentemente do estágio da doença.^(^^5^^)^Em países em desenvolvimento, a maioria dos casos suspeitos não é testada para confirmação do diagnóstico, exceto no caso de pacientes internados. Consequentemente, a taxa de subnotificação é alta.^(^^6^^)^Entretanto, mesmo sem testagem, a identificação clínica de casos suspeitos de COVID-19 é viável e pode ser utilizada para fornecer as devidas orientações a pacientes de baixo risco, a fim de evitar o atendimento presencial no setor de emergência.^(^^7^^)^

Apesar das vantagens teóricas, poucos estudos investigaram o papel da Telemedicina no encaminhamento de pacientes devido a sintomas respiratórios agudos durante a pandemia.^(^^2^^)^A fim de preencher essa lacuna na literatura médica, este estudo se propôs a caracterizar os perfis clínico e demográfico dos pacientes que buscaram atendimento por Telemedicina por conta de sintomas respiratórios agudos durante a pandemia, quantificando os encaminhamentos ao setor de emergência e analisando as variáveis associadas.

## OBJETIVO

Caracterizar as variáveis associadas ao encaminhamento para o setor de emergência mediante consulta por Telemedicina durante a pandemia causada pelo novo coronavírus 2019.

## MÉTODOS

### Desenho experimental e participantes

Este estudo foi aprovado pelo comitê institucional local (CAAE: 31106820.5.0000.0071, parecer 4.001.939). Todos os pacientes acessaram o serviço de Telemedicina do Hospital Israelita Albert Einstein (HIAE), em São Paulo (SP) por meio de um aplicativo para telefone móvel e solicitaram avaliação médica voluntariamente. Entre março e maio de 2020, 500 pacientes adultos (≥18 anos) foram incluídos de forma retrospectiva e aleatória. Foram incluídos os pacientes cujo atendimento foi motivado por sintomas respiratórios de qualquer tipo (tosse aguda ou progressiva, expectoração, dispneia, dor torácica, rinite ou dor de garganta) ou sugestivos de infecção (febre, calafrios, sudorese, mialgia, fadiga, mal-estar, anorexia, dor de cabeça e outros). A suspeita de COVID-19 foi baseada na manifestação de pelo menos um sintoma de cada tipo. O encaminhamento para o setor de emergência foi o critério de avaliação principal. Os pacientes elegíveis foram definidos como os que possuíam dados clínicos completos (impressões subjetivas, queixa e respectiva duração, histórico da doença atual e histórico pessoal), além da descrição dos achados do exame físico. Pacientes com infecção realcionada ao COVID-19 ou com problemas de conexão foram excluídos.

### Escore de Roth

Os pacientes elegíveis foram orientados a realizar o autoexame e a manobra expiratória para obter o escore de Roth. Para o cálculo desse escore, o paciente é instruído a inspirar profundamente e tentar contar até 30 em voz alta, antes de exalar. Após esse procedimento, o paciente é orientado a respirar calma e profundamente por três vezes antes de repetir o teste. O tempo é contado pelo examinador, e o teste válido é o segundo. Neste estudo, foram consideradas três pontuações, de acordo com o número atingido na contagem e o tempo decorrido: normal (>10 e >7 segundos); moderadamente alterado (7 a 10 e/ou 5 a 7 segundos) e gravemente alterado (<7 e/ou <5 segundos). A correlação entre a pontuação no escore de Roth e oximetria corresponde a normal (>95%), moderadamente alterada (90% a 94%) e gravemente alterada (<90%), respectivamente.^(^^8^^)^

### Análise estatística

Este foi um estudo observacional baseado em uma coorte de pacientes aleatoriamente selecionados. A estatística descritiva foi empregada para análise do perfil clínico dos pacientes. As variáveis contínuas foram expressas como medianas e desvio padrão e as categóricas, na forma de contagens e percentagens. Não houve dados faltantes. A regressão logística múltipla e bivariada foi realizada para investigação da correlação entre as variáveis relatadas e coletadas e o critério de avaliação principal. Valores de p<0,05 foram considerados indicativos de diferença significante e o intervalo de confiança (IC) adotado foi de 95%.

## RESULTADOS

Todos os 500 pacientes arrolados no estudo foram analisados. O sexo feminino predominou na amostra (59%), e a média de idade foi baixa (34,7±10,5 anos). Os pacientes buscaram o serviço de Telemedicina, em média, 4,67±4,82 dias após o aparecimento dos sintomas, e quase todos receberam o primeiro atendimento sem ter passado por nenhuma outra consulta virtual ou atendimento presencial no setor de emergência. Quanto às comorbidades preexistentes potencialmente relacionadas a um prognóstico desfavorável para COVID-19, as enfermidades pulmonares crônicas foram as mais prevalentes (8,2%), seguidas por hipertensão (6,2%), obesidade (5,0%), diabetes (2,4% com 0,6% de uso de insulina) e doença cardíaca (2,2%). Um pequeno número de pacientes relatou tabagismo (2,2%), imunossupressão (0,6%) ou doença renal crônica (0,2%). A doença cerebrovascular não foi relatada, e a percentagem de gestantes na amostra estudada foi baixa.

Uma grande percentagem dos pacientes (62,6%) manifestou impressão subjetiva negativa da própria condição clínica, e alguns (41,4%) se autodiagnosticaram como portadores de COVID-19. Apesar da pandemia, quase um quinto dos pacientes autorrelatou resfriado (14,8%), gripe (7,6%) ou pneumonia (2,6%). Alguns relataram suspeita de infecções leves das vias aéreas altas, como sinusite (7,8%) ou faringite (4,4%), e 21,4% não mencionaram nenhum diagnóstico específico. O principal motivo para buscar atendimento por Telemedicina foi o receio de contrair infecção por COVID-19 (43%), seguido do medo de ser encaminhado para o setor de emergência (7,6%). Preocupações relacionadas a internação, transmissão comunitária e ao medo da morte foram relatadas por 6,4%, 6,2% e 0,6% dos pacientes, respectivamente ( Tabela 1 ).

**Tabela 1:** Autoanálise subjetiva dos pacientes

Variável		n (%)
Pergunta: como você se sente?		
	Bem	187 (37,4)
	Mal	313 (62,6)
Pergunta: qual doença você acha que tem?		
	Não sei	107 (21,4)
	COVID-19	207 (41,4)
	Gripe	38 (7,6)
	Resfriado	74 (14,8)
	Pneumonia	13 (2,6)
	Sinusite	39 (7,8)
	Faringite	22 (4,4)
Pergunta: qual seu maior receio quanto à sua condição atual?		
	Nenhum	171 (34,2)
	COVID-19	215 (43)
	Internação	32 (6,4)
	Morte	3 (0,6)
	Exame presencial	38 (7,6)
	Transmissão para outros	31 (6,2)
	Outros	10 (2)

O sintoma respiratório relatado com mais frequência foi a tosse (74,4%), descrita como seca ou produtiva com secreção límpida em 87,4% e 11,2% dos casos, respectivamente. Apenas 1,4% dos pacientes relatou secreção purulenta. A rinorreia foi outro sintoma comum (65,6%), sendo a dor de garganta (38,6%) e o espirro (20,6%) os menos prevalentes. Duas manifestações recentemente relacionadas à infecção por COVID-19 foram descritas: anosmia (24,8%) e disgeusia (24.6%). Dentre os pacientes com sintomas de infecção, 45% tinham febre, 44,8% mialgia ou artralgia, 41,4% fadiga, 40,6% dor de cabeça e 24% sintomas digestivos, como náusea, vômito e diarreia. A dispneia e a dor torácica, sintomas geralmente considerados de maior gravidade, foram relatados por 29,4% e 16% dos pacientes, respectivamente. Metade desses pacientes descreveu a dispneia como leve, e apenas um pequeno número de pacientes relatou dispneia grave ( Tabela 2 ).

**Tabela 2:** Manifestações clínicas

Variável		n (%)
Tosse		372 (74,4)
Muco/secreção		
	Não	437 (87,4)
	Aquoso	56 (11,2)
	Amarelo, verde, marrom	7 (1,4)
	Congestão nasal	217 (43,4)
Rinorreia		
	Não	9 (1,8)
	Sim	328 (65,6)
	Aquosa	157 (31,4)
	Amarela, verde, marrom	6 (1.2)
Espirros		103 (20,6)
Dor de garganta		193 (38,6)
Adenomegalia		14 (2,8)
Rouquidão		45 (9)
Dispneia (subjetiva)		147 (29,4)
Anosmia		124 (24,8)
Disgeusia		123 (24,6)
Intensidade da dispneia subjetiva		
	Não	351 (70,2)
	Leve	73 (14,6)
	Moderada	63 (12,6)
	Grave	13 (2,6)
Dor torácica		80 (16)
Náusea/vômito		45 (9)
Diarreia		75 (15)
Fadiga		207 (41,4)
Mialgia ou artralgia		224 (44,8)
Dor de cabeça		203 (40,6)

No que se refere à possibilidade de realização do exame físico por Telemedicina, além da transmissão de imagem por câmera, alguns pacientes utilizaram dispositivos médicos de uso doméstico para aferição de sinais vitais. Alguns pacientes foram orientados a realizar o autoexame e a manobra expiratória necessária para obter o escore de Roth. Febre foi relatada por 9,4% dos pacientes que acessaram o serviço de Telemedicina, enquanto a sudorese, a inflamação conjuntival e a adenomegalia foram incomuns. A frequência cardíaca foi aferida em 214 pacientes, e a média foi 82,5±14,2. A pressão sanguínea foi aferida em apenas 31 pacientes, e 16 realizaram oximetria – dados que foram de pouca utilidade. O escore de Roth foi calculado em um número expressivo de pacientes (67,6%), obtendo-se resultado normal em 83,5%, moderadamente alterado em 10,7% e gravemente alterado em 5,6% dos casos ( Tabela 3 ). O escore de Roth de todos os 147 pacientes com dispenia autorrelatada foi calculado, e somente em 48 casos (32,6%) houve alterações.

**Tabela 3:** Exame físico

Variável		
Febre		47 (9,4)
Sudorese		4 (0,8)
Adenomegalia detectada por autoexame		12 (2,4)
Inflamação conjuntival		7 (1,4)
Escore de Roth		
	Não realizado	183 (36,6)
	NormaI (nc >10 e tempo >7 segundos)	265/317 (83,5)
	Moderadamente alterado (nc=7-10 e/ou tempo=5-7 segundos)	34/317 (10,7)
	Gravemente alterado (nc <7 e/ou tempo <5 segundos)	18/317 (5,8)
Frequência cardíaca, n=214		82,5±14,2
Pressão arterial sistólica, n=31		134,2±22,4
Pressão arterial diastólica, n=31		86,8±12,6
Oximetria, n=16		96,5±2

Resultados expressos por n (%) ou média±desvio-padrão.

nc: número contado em uma única expiração.

A percentagem de casos suspeitos de COVID-19 foi de 67,6%. Destes, 75% foram conduzidos de forma remota, e exatamente um quarto foi referido para atendimento imediato no setor de emergência. O emprego da Telemedicina aumentou o número de casos suspeitos de COVID-19 em aproximadamente 50% em relação ao autodiagnóstico. As seguintes variáveis mostraram-se independentemente correlacionadas ao encaminhamento para o setor de emergência: sexo feminino (razão de chance *–* RC: 1,91; IC95%: 1,01-3,63; p=0,047), doença cardíaca (RC: 14,28; IC95%: 2,45-83,07; p=0,003), doença pulmonar (RC: 2,49; IC95%: 1,02-6,07; p=0,044), dispneia subjetiva (RC: 3,48; IC95%: 1,76-6,88; p<0,001), dor torácica (RC: 11,34; IC95%: 5,43-23,71; p<0,001), casos suspeitos de COVID-19 (RC: 2,84; IC95%: 1,32-6,09; p=0,007), e escore de Roth moderada ou gravemente alterado (RC: 33,49; IC95% 8,43-133,15; p<0,001) ( Tabela 4 ).

**Tabela 4:** Casos suspeitos de COVID-19 e correlação com encaminhamento

Casos suspeitos de COVID-19, n (%)	338 (67,6)			
Encaminhamento para o setor de emergência, n (%)	125 (25)			
Correlação da variável com encaminhamento para o setor de emergência	RC	IC95%		Valor de p
		Inferior	Superior	
Sexo feminino	1,91	1,01	3,63	0,047
Doença cardíaca	14,28	2,45	83,07	0,003
Doença pulmonar	2,49	1,02	6,07	0,044
Dispneia (subjetiva)	3,48	1,76	6,88	<0,001
Dor torácica	11,34	5,43	23,71	<0,001
Diagnóstico J11 (CID-10)	2,84	1,32	6,09	0,007
Dispneia (escore de Roth)				
Normal	1,86	0,83	4,19	0,132
Moderado a grave	33,49	8,43	133,15	<0,001

Regressão logística bivariada.

RC: razão de chance; IC95%: intervalo de confiança de 95%; CID-10: Classificação Internacional de Doenças.

## DISCUSSÃO

A comunicação virtual representa uma ferramenta multifuncional para lidar com a carga imposta pela COVID-19 sobre o sistema de saúde. Os avanços tecnológicos têm permitido cada vez mais acesso a cuidados de saúde em casos que envolvem separação geográfica entre o paciente e o provedor. A avaliação por áudio e vídeo via *smartphone* vem se tornando cada vez mais viável, o que faz da Telemedicina um recurso de uso potencialmente universal.^(^^9^^)^Além da segurança, do conforto e da facilidade de acesso, o uso dessa ferramenta simples viabiliza a prestação de cuidados de alta qualidade. Principalmente em casos de infecções virais agudas sem tratamento específico instituído, os pacientes podem ser bem avaliados por exame remoto.^(^^1^^,^^2^^)^Ainda assim, apesar das múltiplas vantagens conceituais, a implementação, a regulamentação e a aceitação da Telemedicina por parte da comunidade médica vem sendo lenta, assim como a comprovação de sua efetividade por meio de evidências médicas.^(^^10^^)^Entretanto, a pandemia aumentou a disponibilidade da Telemedicina e desmistificou rapidamente suas limitações, permitindo mais coleta de dados científicos para justificar sua implementação.^(^^11^^)^Em situações de pandemia, o encaminhamento dos pacientes sintomáticos de alto risco para o setor de emergência é fundamental. Evitar que pacientes sem suspeita de COVID-19 e portadores de enfermidades de baixo risco busquem atendimento presencial também é importante para prevenir a contaminação individual e a transmissão comunitária, poupar os profissionais da saúde e evitar o uso inadequado de recursos. O terceiro ponto é o direcionamento correto do tratamento e o gerenciamento da enorme quantidade de notícias instantâneas divulgadas diariamente. A adoção dessas medidas é ainda mais crítica em países em desenvolvimento.^(^^9^^)^

O número de consultas diárias no Departamento de Telemedicina do HIAE aumentou 16 vezes, e a cobertura geográfica também foi expandida. O serviço já vem sendo regulamentado e garante segurança, conforto e bem-estar tanto da equipe médica como dos pacientes. O acesso fácil por aplicativo para telefone móvel permite a realização imediata e barata de consultas médicas 24 horas por dia. Ao final do período de coleta de dados para este estudo, São Paulo tinha 44.411 casos de infecção por COVID-19, 3.600 óbitos, 10 mil internações tinham sido notificadas, e a taxa de ocupação de leitos de unidade de terapia intensiva era de 85%.^(^^12^^)^As limitações intrínsecas da Telemedicina geralmente superestimam o encaminhamento. Qualquer sinal de alerta na anamnese ou exame clínico é digno de investigação presencial. O principal ponto levantado neste estudo é se esse tipo de avaliação médica poderia, de fato, ser uma ferramenta eficaz para diminuir o número de atendimentos de emergência.

Esse estudo foi baseado em uma amostra limitada de 500 pacientes com sintomas respiratórios agudos e sem diagnóstico confirmado de COVID-19. Os pacientes testados com diagnóstico confirmado de COVID-19 foram excluídos, uma vez que já tinham buscado atenção médica e recebido as devidas orientações e acompanhamento. Além disso, Khairat et al., ao analisarem 92 pacientes com diagnóstico confirmado de COVID-19 avaliados por Telemedicina, relataram redução do número de atendimentos no setor de emergência.^(^^13^^)^Do total de pacientes, uma proporção considerável dos que são atendidos em ambulatório (sobretudo os de baixo risco) pode ser tratada a distância de forma eficaz.^(^^14^^)^Em tese, a Telemedicina permite a triagem dos pacientes, evitando o atendimento no setor de emergência. Os pacientes podem ser direcionados para um serviço de atendimento de urgência, acompanhamento médico ambulatorial ou centro local de testagem para COVID-19.^(^^7^^)^Entretanto, não há dados de Telemedicina disponíveis para essa população em particular.^(^^3^^,^^10^^,^^11^^)^

Mann et al., revelaram ampla expansão do atendimento por Telemedicina após o início da pandemia e relataram a realização de 56% de atendimentos de urgência de casos confirmados ou suspeitos de COVID-19, mas não forneceram dados claros sobre o encaminhamento.^(^^10^^)^Assim como no estudo de Khairat et al.,^(^^13^^)^a população investigada neste estudo foi composta principalmente por mulheres adultas jovens. Esse achado pode ter refletido a maior familiaridade dos mais jovens com dispositivos tecnológicos, enquanto pacientes mais velhos, que passam por acompanhamento médico regular, costumam se comunicar com a equipe médica de forma presencial, em vez de recorrer à Telemedicina, ou tendem a buscar atendimento presencial em casos mais graves. A amostra analisada neste estudo foi composta principalmente por pacientes de baixo risco que relataram problemas respiratórios durante a pandemia.

Quase todas as características clínicas da COVID-19 descritas se referem a pacientes hospitalizados (supostamente os casos mais graves), e faltam grandes séries de casos atendidos em ambulatório, sendo que não há descrição de casos envolvendo a Telemedicina.^(^^4^^,^^15^^)^Também não há estudos sobre casos suspeitos de COVID-19 envolvendo pacientes com sintomas respiratórios durante a pandemia. Neste estudo, quase um terço dos pacientes tinha prognóstico desfavorável no que se refere às comorbidades relacionadas à infecção por COVID-19. Segundo as diretrizes de saúde, esses pacientes devem buscar atenção médica. Além disso, os 62,6% de pacientes que classificaram subjetivamente a própria condição geral de saúde como ruim supostamente buscariam serviços de saúde. Esse achado já basta para corroborar o benefício da avaliação inicial por de Telemedicina. A anamnese revelou ampla gama de sintomas respiratórios, e a equipe médica suspeitou de COVID-19 em 67,6% dos casos, aumentando o índice de autodiagnóstico. Independente do encaminhamento para o setor de emergência, a maioria dos pacientes já tinha se beneficiado de orientações comportamentais para casos suspeitos de COVID-19. A dor torácica e a dispneia foram relatadas por 16% e 29,4% dos pacientes, respectivamente. O fato de que sintomas desse tipo são muito mais difíceis de avaliar por Telemedicina faz da dor torácica um dos principais determinantes do encaminhamento para o setor de emergência.

Cada vez mais tentativas vêm sendo feitas para expandir a condução de exames físicos à distância. Na dermatologia, na oftalmologia e na neurologia, o tele-exame pode ser facilmente realizado.^(^^16^^)^A Telemedicina permite a observação de corrimento nasal e tosse (seca ou produtiva), além do exame do estado mental, expressões faciais de dor, prostração e sudorese, hiperemia, cianose ou palidez cutânea. Eventualmente, chiados no peito podem ser detectados. Angústia e frequência respiratórias, fala interrompida, uso da musculatura acessória, batida de asa do nariz e respiração paradoxal são relativamente fáceis de identificar. Os sinais vitais podem ser aferidos por autoexame (guiado ou não) e por meio de dispositivos domésticos (termômetro, esfigmomanômetro, relógio e outros medidores de frequência, oxímetro e aplicativos para telefones móveis). O exame da orofaringe pode ser viabilizado pelo posicionamento correto da câmera e permite a detecção de eritema, exsudato e alterações nas amigdalas. Linfonodos aumentados podem ser palpados mediante autoexame guiado pelo médico.^(^^2^^,^^17^^)^Entretanto, o ponto principal é a avaliação do comprometimento pulmonar. A dispneia é um sintoma associado a doenças potencialmente fatais.^(^^18^^)^Este estudo revelou associação significante entre a dispneia e o encaminhamento para o setor de emergência. Entretanto, a definição de dispneia é subjetiva, e a patofisiologia multifatorial dificulta a determinação da gravidade do quadro e do diagnóstico etiológico. Uma revisão sistemática concluiu que não há padrão-ouro para a avaliação clínica da dispneia, o que torna os exames adicionais mandatórios.^(^^19^^)^Na Telemedicina, a avaliação da dispneia se torna ainda mais complexa. Com base na observação clínica, a maioria dos pacientes não apresentava dispneia, porém isso não descarta distúrbios de oxigenação ou ventilação. Poucos pacientes utilizaram a oximetria. Nesta amostra, 317 pacientes foram avaliados com base no escore de Roth (contagem e/ou duração de uma única respiração), que pode ser associado a diferentes valores de corte de oximetria de pulso. Esse escore viabiliza o uso da Telemedicina para determinação da ocorrência e da possível quantificação da dispneia. O escore de Roth costuma ser aplicado em casos de dispneia ou sinais de alerta identificados no exame clínico. De 317 pacientes, 83,6% apresentavam valores expiratórios normais e foram tratados de forma conservativa. Em contrapartida, escores moderada ou gravemente alterados mostraram-se associados, de modo significativo, ao encaminhamento para o setor de emergência. A aplicação do escore de Roth permitiu a reclassificação de quase dois terços dos pacientes com dispneia autorrelatada. Portanto, o escore de Roth mostrou-se associado a mudanças de conduta médica na maioria dos casos em que sua aplicação foi necessária.

Neste estudo, exatamente um quarto dos pacientes foi encaminhada para o setor de emergência, enquanto 75% foram tratados de forma remota. Os casos julgados suspeitos de COVID-19 pela equipe médica mostraram-se relacionados ao encaminhamento para o setor de emergência, a fim de garantir o acesso a tratamento para condições potencialmente mais graves. As comorbidades graves (doenças pulmonares e cardíacas crônicas) e a dor torácica também se mostraram associadas ao encaminhamento – as primeiras por guardarem relação com a predisposição à infecção grave, e a segunda por ser uma possível manifestação de evento trombótico ou complicação pulmonar.^(^^4^^,^^14^^)^Esses achados sugerem que pacientes de alto risco podem de fato ser identicados facilmente por exame remoto. Os demais pacientes representam o maior desafio a ser vencido pela Telemedicina. Em que pese o alto número de pacientes que se autoperceberam como graves, a anamnese e o exame físico (incluindo o escore de Roth) permitiram sua reclassificação como de baixo risco. A dispneia autorrelatada também foi um fator associado ao encaminhamento e representa uma limitação do exame físico remoto, embora alguns pacientes dispneicos tenham deixado de ser encaminhados com base no julgamento clínico e no escore de Roth. Escores normais desencorajaram o encaminhamento, e apenas alguns pacientes com alterações moderadas a graves foram encaminhados.

Algumas soluções para o enfrentamento dos desafios sem precedentes introduzidos pelo surto de uma doença infecciosa devem ser incorporadas na Telemedicina.^(^^1^^)^O impacto do surto de COVID-19 no sistema de saúde italiano talvez tivesse sido menor se a Telemedicina tivesse sido amplamente implementada no país. A percepção subjetiva foi de que não houve resistência por parte dos pacientes.^(^^10^^,^^14^^)^Além disso, considerando a possibilidade de esgotamento ( *burnout* ) dos prestadores de cuidados de saúde em resposta à grande carga imposta pela COVID-19, a implementação da Telemedicina pode garantir a eficiência do sistema de saúde.^(^^20^^)^

Este é um momento único para o cuidado virtual de saúde, e, possivelmente, a maior transformação jamais vista nesse setor no âmbito global se dará em um futuro próximo. Além do conforto e da segurança proporcionados por esse tipo de serviço, há evidências crescentes da efetividade da Telemedicina no encaminhamento de pacientes de alto risco e na orientação de pacientes de baixo risco. Com exceção do exame físico, a Telemedicina deve proporcionar qualidade de cuidado equivalente ao atendimento presencial.^(^^17^^)^Diversas estratégias de exame físico a distância podem agregar valor à anamneses, e, eventualmente, testemunharemos o surgimento de dispositivos médicos de uso doméstico capazes de transmitir informações relevantes. Este estudo demonstrou que a Telemedicina foi capaz de encaminhar corretamente os pacientes de alto risco e reclassificar e redirecionar os pacientes de baixo risco durante uma pandemia, reduzindo a busca voluntária por atendimento de emergência.

Entretanto, este estudo tem algumas limitações. Em primeiro lugar, trata-se de um estudo observacional e retrospectivo. Em segundo lugar, não houve confirmação posterior do diagnóstico de COVID-19. Em terceiro lugar, embora o escore de Roth tenha sido utilizado com frequência, não há validação externa da correlação com a dessaturação. Finalmente, não houve seguimento após a recomendação de encaminhamento. Em termos de pontos fortes, este estudo reflete um cenário real de prática médica, no qual 500 pacientes buscaram o serviço de atendimento virtual por uma condição muito comum de saúde.

## CONCLUSÃO

No que se refere a pacientes com sintomatologia respiratória aguda durante a pandemia, a avaliação por Telemedicina possibilitou a reclassificação da impressão subjetiva dos pacientes, além de uma melhor inspeção da infecção causada pela COVID-2019 e a identificação de pacientes de risco. A realização do exame médico virtual otimizou o encaminhamento, evitando o atendimento presencial inadequado e permitindo a devida orientação dos pacientes de baixo risco. A aplicação remota do escore de Roth deve ser encorajada. A Telemedicina pode ser incorporada ao sistema de saúde como ferramenta estratégica com boa relação custo-benefício para o atendimento inicial de pacientes agudos.
